# Criterion Validity of the 30‐Second Chair Stand Test for Lower‐Limb Muscle Strength in Adults With Severe Mental Disorders: The PsychiActive Project

**DOI:** 10.1002/pri.70344

**Published:** 2026-09-09

**Authors:** Alvaro Lopez‐Moral, Diego Munguia‐Izquierdo, Javier Bueno‐Antequera

**Affiliations:** ^1^ Physical Performance & Sports Research Center (CIRFD), Department of Sports and Computer Science, Section of Physical Education and Sports, Faculty of Sports Sciences Universidad Pablo de Olavide Seville Spain; ^2^ Department of Physical Education and Sport Universidad de Sevilla Seville Spain; ^3^ Research Group in Development Movimiento Humano Universidad de Zaragoza Zaragoza Spain

**Keywords:** chair stand test, functional assessment, muscle strength, physical function, physical performance, rehabilitation, severe mental disorders

## Abstract

**Background and Purpose:**

Individuals with Severe Mental Disorders (SMDs) face a high risk of premature mortality and disability due to accelerated neuromuscular decline. Despite the critical need to monitor physical function, the lack of validated, time‐efficient tools limits their clinical utility and hinders the integration of objective assessments into routine care. This gap limits the early identification of physical function impairments and the implementation of timely evidence‐based interventions. This is the first study to evaluate the criterion validity of the 30‐s Chair Stand Test (30s‐CST) as a pragmatic indicator of lower‐limb muscle strength in adults with SMDs, providing a feasible functional indicator to support routine clinical assessment.

**Methods:**

Using a cross‐sectional validation design (STROBE), this study was conducted with 91 adults (18–65 years) diagnosed with SMDs. Participants performed the 30s‐CST and reference isometric Knee Extension Strength (KES) testing. Statistical analyses included Pearson's correlation (*r*), Intraclass Correlation Coefficients (*ICC*), and Bland–Altman plots to determine agreement and heteroscedasticity, with sub‐analyses by sex, age, and body mass index.

**Results:**

Analysis revealed significant moderate correlation and agreement (*r* = 0.56 [95% CI 0.40–0.69]; ICC = 0.54 [0.38–0.67]; both *p* < 0.001) for the total sample, with stronger results in the female subgroup. The 30s‐CST demonstrated precision, with a prediction error lower than the within‐subject variability of the reference method. Bland–Altman plots showed acceptable agreement, with narrow limits of agreement (−1.79–1.80), and analysis indicated homoscedasticity.

**Discussion:**

The 30s‐CST appears to be a pragmatic and time‐efficient screening tool for identifying individuals with reduced lower‐limb muscle strength in SMDs. Although limited by the lack of biomechanical specificity between dynamic and isometric methods, the test offers a balance between clinical feasibility and its ability to provide a functional estimate related to lower‐limb muscle strength. Integrating this pragmatic screening tool into routine practice may support the assessment of functional performance in resource‐constrained settings where more complex strength assessments are not readily available.

## Highlights

- What is known?
  ◦ Patients with severe mental disorders (SMDs) experience accelerated functional decline and reduced muscle strength, increasing disability and mortality risk. Despite its clinical importance, muscle strength assessment is rarely integrated into routine psychiatric care because of a lack of time‐efficient, low‐cost, and feasible functional tools suitable for clinical settings.
- What is new?
  ◦ This is the first study to provide evidence of the criterion validity of the 30‐s Chair Stand Test (30s‐CST) as a pragmatic indicator of lower‐limb muscle strength in individuals with SMDs, a population in which its criterion validity had not previously been established. Compared with validated clinical isometric assessments, the 30s‐CST may provide clinically useful information for routine screening. This functional indicator may complement the assessment of lower‐limb muscle strength in routine clinical practice.

## 1 Introduction

Severe mental disorders (SMDs), including schizophrenia, bipolar disorder, schizoaffective disorder, and major depression, are among the leading causes of disability and premature mortality worldwide, with no evidence of a reduction in their global burden since 1990 (GBD 2019 Mental Disorders Collaborators 2022; Walker et al. 2015). Beyond their impact on health, SMDs also impose a considerable economic burden, which in 2010 was estimated to be comparable to that of cardiovascular diseases and greater than that of cancer, chronic respiratory diseases, and diabetes, with costs projected to double by 2030 (Trautmann et al. 2016). As a result, improving the prevention and treatment of SMDs has become a major public health priority (Wykes et al. 2015).

Improving health assessment may help prioritize interventions and reduce the growing burden associated with SMDs by identifying individuals who require timely and targeted care. Among the different indicators of physical health, muscle strength (MS) has emerged as a robust predictor of adverse outcomes, with low MS consistently associated with an increased risk of all‐cause mortality and cancer in both healthy and clinical populations, independent of age, body fat, smoking, alcohol consumption, and hypertension (Artero et al. 2012; Celis‐Morales et al. 2018; García‐Hermoso et al. 2018; Jochem et al. 2019; Volaklis et al. 2015). This issue is particularly relevant for people with SMDs, who consistently exhibit lower MS than the general population (Nygård et al. 2019; Vancampfort et al. 2016). Therefore, routine assessment of MS could facilitate the screening of vulnerable individuals while enabling the monitoring of an important health outcome. However, healthcare staff often overlook its use in clinical settings, particularly in low‐income countries, due to resource constraints, time limitations, and inadequate training. Although functional physical performance tests may provide a feasible alternative for estimating MS in clinical settings, no such test has yet been validated for people with SMDs (de Oliveira Tavares et al. 2021).

The 30‐s Chair Stand Test (30s‐CST) is a simple and time‐efficient functional test that uses a chair, a stopwatch, and an evaluator to provide an indirect indicator of lower‐limb MS. It has been widely applied in both healthy (Tveter et al. 2014; Rikli and Jones 2013) and clinical populations (Santos E Campos et al. 2020; Nygård et al. 2021), offering a practical alternative to more complex assessments that require specialized equipment. Despite its widespread use, criterion validity against a reference measure of MS has only been examined in three studies, all conducted in healthy adults older than 60 years (Rikli and Jones 1999; Macfarlane et al. 2006; McCarthy et al. 2004). To date, this measurement property has not been evaluated in individuals with SMDs. In addition, previous studies assessed validity exclusively using Pearson's correlation coefficient (*r*), without reporting the intraclass correlation coefficient (ICC), which provides complementary information on agreement between methods (Bland and Altman 1986a; Lin 1989). Therefore, further studies are needed to establish the criterion validity of the 30s‐CST in populations where this property remains unexplored, including individuals with SMDs, by evaluating its agreement with a reference method.

The primary aim of this study was to assess the criterion validity of the 30s‐CST as a pragmatic indicator of lower‐limb MS in individuals with SMDs while examining the potential influence of age, sex, and body mass index (BMI) on its validity. We hypothesized that the 30s‐CST would demonstrate low‐to‐moderate criterion validity for estimating MS across these subgroups. This hypothesis was based on the findings of McCarthy et al. (2004), who reported a low correlation between 30s‐CST performance and MS measured by hand‐held dynamometry in healthy older adults.

## 2 Methods

### 2.1 Study Design and Participants

Between March 2018 and July 2022, a convenience sample of adults with SMDs, including schizophrenia, schizoaffective disorder, bipolar disorder, major depressive disorder, personality disorders, and severe stress‐related disorders according to the International Classification of Diseases, 10th Revision (ICD‐10), receiving stable pharmacological treatment (i.e., no treatment changes during the previous month), was recruited from seven outpatient mental health services in southern Spain. Recruitment was temporarily suspended during the COVID‐19 restrictions. No changes were made to the study protocol or assessment procedures following the resumption of recruitment. Exclusion criteria included clinical instability (defined as the presence of acute psychiatric symptoms, recent changes in clinical status within the previous 3 months, or treatment changes within the previous month), comorbid substance abuse, and evidence of uncontrolled cardiovascular, neuromuscular, and endocrine disorders. Details of the recruitment procedures, eligibility criteria, and general assessment protocol have been described previously (Lopez‐Moral et al. 2025, 2026). Participants were grouped under the clinical construct of SMDs because, despite diagnostic differences, they share common physical health impairments and rehabilitation needs. Furthermore, this approach reflects the heterogeneous case‐mix typically encountered in routine outpatient mental health services, thereby enhancing the clinical applicability of the findings.

Anthropometric and physical assessments were completed during a single 30‐min session by the same evaluator, an exercise physiologist with more than six years of experience. Assessments were performed in a standardized order, beginning with anthropometric measurements, followed by the 30s‐CST and the knee extension strength (KES) test, the latter being considered a maximal effort assessment. Information on age and diagnosis was extracted from participants' medical records. Psychiatric symptom severity during the previous week was assessed using the Brief Symptom Inventory‐18, with scores ranging from 0 to 72, where higher scores indicate greater symptom severity (Derogatis 2001).

All procedures complied with the ethical standards of the relevant institutional and national committees on human experimentation and with the Declaration of Helsinki (1975, revised in 2013). The study was approved by the ethics committees of the Virgen Macarena and Virgen del Rocio University Hospitals (1674‐N‐17) and was conducted in accordance with the STROBE (STrengthening the Reporting of OBservational Studies in Epidemiology) guidelines. Written informed consent was obtained from all participants before enrollment, and no financial compensation was provided.

### 2.2 Anthropometric Measurement

Anthropometric measurements followed procedures described previously (Lopez‐Moral et al. 2025, 2026). Height was measured using a stadiometer (Seca 711, Hamburg, Germany), and body weight was assessed using an InBody 770 device (Biospace, Seoul, South Korea). Each measurement was performed twice, and the mean value was used for subsequent analyses. Body mass index (BMI) was calculated as body weight (kg) divided by height squared (m^2^). All assessments were performed under standardized conditions following a minimum three‐hour fast and abstention from fluid intake. Participants were instructed to wear light clothing, remove all metal objects, and empty their bladders before testing. Measurements were conducted in a ventilated examination room under controlled temperature and humidity conditions.

### 2.3 Measurement of 30‐Second Chair Stand Test (30s‐CST)

The 30‐Second Chair Stand Test (30s‐CST) was performed according to the protocol described by Rikli and Jones (2013). A standardized chair with a fixed height of 43 cm, without armrests and not supported against a wall, was used for all assessments. Participants started from a seated position in the middle of the chair with their back upright, feet shoulder‐width apart, and arms crossed over the chest with the palms facing inward. Following a verbal signal, they were instructed to stand up to full knee extension and sit down as many times as possible within 30 seconds. A pre‐demonstration was performed, and the participants completed two familiarization repetitions before testing. A repetition was considered valid when full knee extension was achieved while maintaining an upright and stable posture. A multimedia demonstration of the test protocol is available at https://upotv.upo.es/video/58da21db238583e0478b48f3. During the test, the evaluator stood beside the participant to ensure correct execution, count valid repetitions, and provide standardized verbal encouragement. Only one test trial was performed, and the outcome was the total number of valid repetitions completed within 30 seconds.

### 2.4 Measurement of Isometric Knee Extension Strength (KES)

Isometric KES was measured using a Lafayette Manual Muscle Testing System Model‐01165 hand‐held dynamometer (Lafayette Instrument Company, Lafayette IN, USA) (Bohannon 1997), which has demonstrated very strong criterion validity against established measures of lower‐limb strength (1RM, *r* = 0.82) (Tan et al. 2018). The hand‐held dynamometer was selected as the reference method because of its established validity, reliability, portability, and feasibility for use in routine clinical practice (Stark et al. 2011), particularly in mental health services where laboratory‐based equipment is often unavailable. The procedures used to assess KES have been described previously (Lopez‐Moral et al. 2025) and were based on the protocol proposed by Mentiplay et al. (2015). Briefly, participants were seated with their hips and knees flexed at 90° and their hands placed on either side of the stretcher for stabilization. The dynamometer was positioned on the anterior aspect of the tibia, proximal to the ankle joint. To minimize evaluator interference during testing, the stretcher was placed close to a wall, allowing the evaluator to stabilize their arm and counteract the force generated by the participant. Three five‐second maximal attempts were performed alternately with each leg, separated by 60‐s rest intervals to minimize fatigue. Standardized verbal encouragement was provided during each attempt. Following previous validity studies (Rikli and Jones 1999; Macfarlane et al. 2006; McCarthy et al. 2004), the highest body weight‐adjusted value (*N*/kg) obtained across all attempts was used for the analysis.

### 2.5 Data Analysis

Participants with a complete and valid dataset of anthropometric variables, KES and 30s‐CST were included in the analysis. Because the two methods were expressed in different units (repetitions and *N*/kg), sex‐specific standardized z‐scores were calculated before performing the agreement analysis. This transformation expressed both measures on a common dimensionless scale while preserving the relative standing of each observation within its distribution (Altman 1991; Cohen 1988), thereby facilitating agreement analysis between methods expressed in different measurement units. Agreement between the standardized measures was evaluated using the intraclass correlation coefficient (ICC) (Bland and Altman 1990; Lee et al. 1989) calculated using a two‐way mixed‐effects model with a consistency definition, together with its 95% confidence interval (95% CI). The ICC was selected because, unlike Pearson's correlation coefficient, it evaluates the degree of agreement between measurements rather than only their association. ICC values were interpreted as poor (< 0.50), moderate (0.50–0.75), good (0.75–0.90), or excellent (> 0.90) (Koo and Li 2016). The standard error of estimate (SEE) was calculated to assess the precision of individual scores. Pearson's correlation coefficient (*r*) was also reported to facilitate comparisons with previous criterion validity studies of the 30s‐CST. According to Mukaka (Mukaka 2012), *r* values between 0.3 and 0.5, 0.5 and 0.7, and 0.7 and 0.9 were interpreted as low, moderate, and high correlations, respectively. Agreement was further examined using Bland–Altman plots (Bland and Altman 1986b), including 95% limits of agreement (LOA). Heteroscedasticity was assessed by examining the association between measurement differences and their magnitude using linear regression analysis. Subgroup analyses were conducted according to sex, age, and BMI, given their known influence on MS and 30s‐CST performance (Tveter et al. 2014). Age and BMI subgroups were defined using the sample median as the cut‐off value. The data were analyzed using IBM's SPSS (Version 25; IBM Corp., Armonk, NY, USA) with a significance level of *p* < 0.05.

## 3 Results

A total of 119 individuals were screened for eligibility. Of these, 91 were included in the analysis, and 28 were excluded (7 due to age > 65 years and 21 due to incomplete assessment data). Table 1 presents the descriptive characteristics of 91 participants, stratified by sex (63 men, 28 women); the mean age was 39.7 ± 9.4 years (range: 18–65 years). The predominant diagnoses were schizophrenia, schizotypal, and delusional disorders (61.5%), followed by mood disorders (19.8%).

**TABLE 1:** Sociodemographic and clinical characteristics of the study participants.

	All (*n* = 91)	Men (*n* = 63)	Women (*n* = 28)
Age (years)	39.7 ± 9.4 [18–65]	38.3 ± 8.6 [18–60]	42.9 ± 10.5 [20–65]
Height (cm)	172.2 ± 9.0	176.4 ± 6.2	162.7 ± 6.6
Weight (kg)	91.8 ± 21.5	95.5 ± 19.3	84.5 ± 24.3
BMI (kg/m^2^)	30.9 ± 6.6	30.5 ± 5.7	31.8 ± 8.2
Illness duration (years)	13.0 ± 9.3	12.0 ± 9.1	14.7 ± 9.7
Diagnoses^a^			
Schizophrenia, schizotypal and delusional disorders	56 (61.5%)	45 (71.4%)	11 (39.3%)
Mood [affective] disorders	18 (19.8%)	11 (17.5%)	7 (25.0%)
Neurotic, stress‐related and somatoform disorders	3 (3.3%)	1 (1.6%)	2 (7.1%)
Disorders of adult personality and behavior	14 (15.4%)	6 (9.5%)	8 (28.6%)

*Note:* Values expressed as mean ± SD, frequency (%), and range [min–max].

^a^ Diagnoses consistent with the ICD‐10 categories for SMDs.

Table 2 summarizes the agreement between the 30s‐CST and the KES tests across the whole sample, and all analyzed subgroups. Moderate Pearson's correlation coefficients and ICC values were observed for the whole sample (*r* = 0.56 [95% CI 0.40–0.69]; ICC = 0.54 [0.38–0.67]; both *p* < 0.001), whereas the highest correlation and agreement were found in women (*r* = 0.73 [95% CI 0.49–0.87]; ICC = 0.73 [0.49–0.86]). Significant correlations were observed across all subgroups (all *p* < 0.05), and the SEE of the 30s‐CST was lower than the within‐subject variability (SD) of the KES test.

**TABLE 2:** Analysis of agreement and correlation between 30s‐CST and KES tests.

		30s‐CST		KES		KES/BW					
	*N*	Mean	SD	Mean	SD	Mean	SD	ICC (95% CI)	SEE	*r* (95% CI)	*p*‐value
All	91	22.3	6.3	412.5	145.1	4.6	1.5	0.54 (0.38–0.67)	137.1	0.56 (0.40–0.69)	< 0.001
Gender											
Men	63	23.0	0.7	466.0	130.6	5.0	1.3	0.45 (0.23–0.63)	129.0	0.45 (0.23–0.63)	< 0.001
Women	28	20.4	1.2	292.1	96.4	3.7	1.8	0.73 (0.49–0.86)	83.5	0.73 (0.49–0.87)	< 0.001
Age (years)											
18–40	48	24.6	5.8	445.5	117.9	5.0	1.2	0.45 (0.19–0.65)	119.1	0.41 (0.14–0.62)	< 0.001
41–65	43	19.3	5.5	372.2	163.2	4.2	1.7	0.58 (0.34–0.75)	146.3	0.61 (0.38–0.77)	0.002
BMI category											
Non‐obese (BMI < 30)	44	20.5	5.6	445.7	165.9	4.2	1.5	0.49 (0.24–0.68)	147.9	0.52 (0.26–0.71)	< 0.001
Obese (BMI ≥ 30)	47	24.1	6.4	377.0	110.2	5.0	1.3	0.51 (0.25–0.70)	103.0	0.54 (0.30–0.72)	< 0.001

Abbreviations: 30s‐CST, 30‐s Chair Stand Test (number of repetitions); BMI, body mass index (kg/m^2^); BW, body weight (kg); CI, confidence interval; ICC, intraclass correlation coefficient; KES, knee extension strength (N); *r*, Pearson correlation coefficient; SD, standard deviation; SEE, standard error of estimate.

**FIGURE 1:**
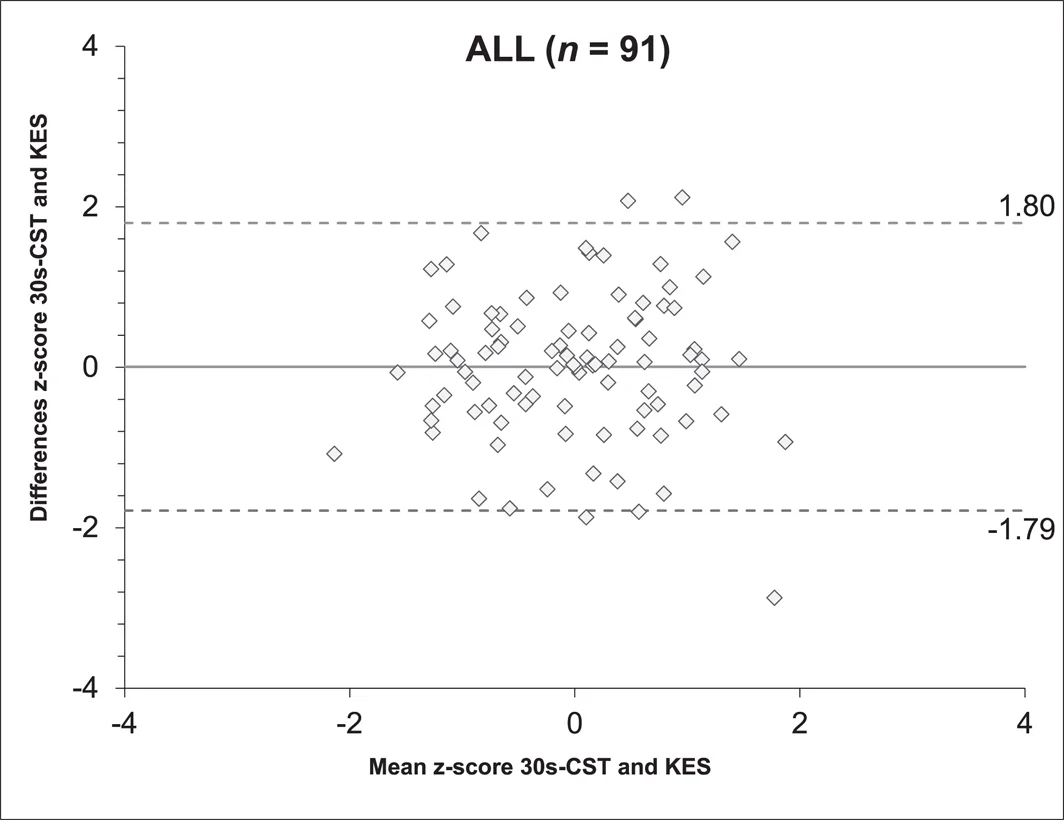
Bland–Altman plots with limits of agreement (LOA) of z‐scores for the 30s‐CST and KES tests in adults with SMDs. The thick middle line represents the mean difference between the z‐scores of the 30s‐CST and KES tests, while the dashed lines represent the upper and lower 95% LOA (mean difference ± 1.96 SD).

Figure 1 illustrates the Bland–Altman plots and the LOA values ranging from −1.79 to 1.80 for the entire sample. No significant association was observed between the differences and the magnitude of MS (*β* = 0.09; *p* = 0.930), indicating homoscedasticity. The narrowest LOA values were identified in the women group, ranging from −1.43 to 1.49 (Supporting Informatio S1: Figure S1). Differences between methods also remained consistent across all subgroups analyzed (*β* = −0.219 to 0.086; Ps = 0.143–0.950).

## 4 Discussion

The present study demonstrated moderate criterion validity of the 30s‐CST as a pragmatic indicator of lower‐limb MS in individuals with SMDs. Analyses of the Bland–Altman and heteroscedasticity plots indicated that, regardless of the magnitude of MS, the differences between the 30s‐CST and KES tests did not increase, supporting the consistency of the agreement across the range of observed values. Moreover, the SEE was lower than the within‐subject variability (SD) of the reference method, indicating that the prediction error was smaller than the natural variability of the reference measure and supporting the precision of the 30s‐CST for its intended use as a pragmatic screening tool in routine clinical practice. These findings, together with the inclusion of a heterogeneous clinical sample representative of routine outpatient mental health services and the analysis of age, sex, and BMI subgroups, provide further support for the use of the 30s‐CST as a feasible screening indicator of lower‐limb MS in routine clinical settings involving individuals with SMDs.

To facilitate the clinical interpretation of the 30‐s Chair Stand Test (30s‐CST), our findings can be considered alongside preliminary population‐specific reference values recently reported for individuals with SMDs in a Research Square preprint (not yet peer reviewed) (Bueno‐Antequera et al. 2025). In that study, performance below the 25th percentile was proposed as a threshold to identify individuals at increased risk of impaired physical fitness. Although the present study was not designed to establish diagnostic cut‐off values, the evidence provided here supports the clinical applicability of the 30s‐CST as a pragmatic screening indicator of lower‐limb MS. Together, these findings suggest that preliminary population‐specific reference values may facilitate the implementation of the 30s‐CST in routine practice for functional assessment and monitoring functional status.

To our knowledge, this is the first study to examine the criterion validity of the 30s‐CST in individuals with SMDs. Therefore, our findings should be interpreted in the context of the limited evidence available from other populations. To date, only three studies have evaluated the criterion validity of the 30s‐CST, all of which were conducted in healthy adults aged over 60 years. Macfarlane et al. (2006) and McCarthy et al. (2004) reported lower correlations (*r* = 0.24 and *r* = 0.44, respectively), whereas Rikli and Jones (1999) found a stronger association (*r* = 0.77) than that observed in the present study (*r* = 0.56). These differences may be explained, at least in part, by variations in age and baseline MS across study populations. As described by Samson (2000), MS, particularly knee extension strength, declines more rapidly from approximately 55 years of age, with a steeper reduction in women than in men. Given that the 30s‐CST is influenced by lower‐limb MS, these age‐related changes may have influenced the strength of the association observed between the 30s‐CST and the reference measure. In this regard, the studies by Macfarlane et al. (2006) and McCarthy et al. (2004) included exclusively older adults, who would be expected to present lower levels of MS, which may partly explain the weaker associations reported between 30s‐CST performance and KES. In contrast, the participants included by Rikli and Jones (1999) were younger overall, as individuals older than 90 years were excluded, and they also achieved higher baseline 30s‐CST scores than those reported by Macfarlane et al. (2006). Taken together, these differences in participant characteristics may partly explain the variability in validity reported across studies and highlight the importance of considering age when interpreting the validity of the 30s‐CST.

Another factor that may explain the differences in the criterion validity of the 30s‐CST across studies is the use of different measurement protocols. The correlation observed in the present study was higher than that reported by Macfarlane et al. (2006) (*r* = 0.56 vs. *r* = 0.24), despite both studies comparing the 30s‐CST with KES measured using a hand‐held dynamometer. Several methodological differences may have contributed to this discrepancy. In the present study, the 30s‐CST was administered by an experienced evaluator who ensured correct execution of the test, recorded only valid repetitions, and provided standardized verbal encouragement throughout the assessment. In contrast, participants in the study by Macfarlane et al. (2006) performed the test unsupervised in their homes. Furthermore, a standardized chair without armrests and with a fixed height (43 cm) was used to minimize the influence of chair characteristics on performance (Kuo 2013), whereas chair height could not be standardized under the home‐based conditions of the previous study. Participants also received a demonstration of the test and completed two familiarization repetitions before the assessment. Likewise, the KES protocol was standardized by controlling participant positioning, evaluator positioning, and testing conditions, with both assessments performed consecutively by the same evaluator. As several of these methodological details were not reported by Macfarlane et al. (2006), differences in testing procedures may have contributed to the variability observed between studies. This explanation is further supported by the findings of Rikli and Jones (1999) and McCarthy et al. (2004), who both followed the standardized 30s‐CST protocol proposed by Rikli and Jones (2013) and, despite using different reference methods, reported stronger correlations than those observed by Macfarlane et al. (2006). McCarthy et al. (2004) found a correlation (*r* = 0.44) comparable to that of the present study, using isokinetic knee extension strength as the reference measure, whereas Rikli and Jones (1999) reported a higher correlation (*r* = 0.77) when lower‐limb strength was assessed dynamically using a leg press. These differences may partly reflect the influence of the reference method selected to evaluate the criterion validity of the 30s‐CST. Unlike isometric or isokinetic knee extension tests, which quantify maximal force under fixed or constant‐velocity conditions, the 30s‐CST evaluates performance during a dynamic functional movement. Consequently, its performance reflects not only lower‐limb MS but also other components of physical function, including movement coordination, balance, movement speed, and muscular endurance. Therefore, only moderate agreement between both methods would be expected despite their conceptual relationship (Schell and Leelarthaepin 1994). Accordingly, the moderate agreement observed in the present study should be interpreted as reflecting the different constructs assessed using the two methods rather than the limitations of either assessment.

Finally, the stronger correlation and agreement observed in the women subgroup deserve further consideration. Sex‐specific differences in 30s‐CST performance and lower‐limb muscle function have previously been reported, suggesting that the relationship between functional performance and MS may differ between men and women (Alcazar et al. 2021). However, no formal statistical comparisons between subgroup correlations or ICC values were performed in the present study. Therefore, these findings should be considered exploratory and require confirmation in larger studies specifically designed to investigate sex‐related differences in the criterion validity of the 30s‐CST.

Overall, our findings are consistent with previous validation studies (Tveter et al. 2014; Rikli and Jones 2013) and extend the available evidence to adults with SMDs. Although agreement with the reference measure was moderate, the 30s‐CST appears to be a feasible and pragmatic screening indicator related to lower‐limb MS in this population. Its simplicity, low cost, and ease of implementation make it a feasible option for routine clinical settings where laboratory‐based assessments are not readily available. Future longitudinal studies should validate population‐specific cut‐off values, evaluate screening performance, determine responsiveness, and establish the ability of the 30s‐CST to predict clinically relevant outcomes.

## 5 Limitations

One of the main limitations of this study relates to the devices and protocols used to measure lower‐limb MS. The methods employed yielded different units of measurement; therefore, standardized z‐scores were used for all analyses. In addition, the conceptual differences between the 30s‐CST test, which evaluates performance during a dynamic functional task, and the KES test, which measures maximal isometric strength, may have influenced the observed level of agreement. Future validation studies should therefore consider criterion methods involving movement patterns and speeds that more closely resemble those of the 30s‐CST (Schell and Leelarthaepin 1994). Additionally, potential confounding factors, such as joint range of motion, balance, sensorimotor function, physical activity levels, duration of illness, psychiatric symptom severity, and antipsychotic medication‐related effects (e.g., sedation or extrapyramidal symptoms), were not included in the statistical analyses (Lord et al. 2002; Schenkman et al. 1996). Furthermore, lower‐limb musculoskeletal conditions that did not preclude safe test performance were not systematically recorded or considered in the analyses and therefore may also have influenced 30s‐CST performance. Future studies should use multivariable approaches to investigate the influence of these factors on 30s‐CST performance in individuals with SMDs. Another limitation is that the responsiveness of the 30s‐CST, which is relevant for determining its ability to detect meaningful changes in functional performance over time or following specific interventions, was not assessed. Furthermore, this study was designed to evaluate criterion validity rather than screening performance. Consequently, diagnostic accuracy metrics, such as sensitivity, specificity, predictive values, or the area under the receiver operating characteristic curve, were not assessed. Future studies should externally validate recently proposed population‐specific cut‐off values and determine the diagnostic accuracy of the 30s‐CST for identifying individuals with reduced lower‐limb strength‐related functional performance and other clinically relevant outcomes. No a priori sample size calculation was performed because this study used a convenience sample. Although the overall sample size was adequate for the primary criterion validity analyses, the women subgroup (*n* = 28) was relatively small. Therefore, subgroup findings should be considered exploratory and interpreted with caution until confirmed in larger studies. Moreover, the generalizability of these findings may be limited, as participants were recruited from seven outpatient mental health services within a single region of Spain and were clinically stable at the time of assessment. Therefore, caution is warranted when extrapolating these results to individuals experiencing acute psychiatric episodes, inpatient settings, or older populations. Future multicenter studies are needed to externally validate these findings. Finally, the predominance of male participants may limit the interpretation of the findings across the broader SMD population, although results were presented separately by subgroup to facilitate the interpretation of potential differences according to age, sex, and BMI.

Despite these limitations, this study included complementary measures of agreement, correlation, and measurement precision, which contributed to a better interpretation of the results and addressed several methodological limitations of previous studies. This study is the first to assess the criterion validity of the 30s‐CST in adults with SMDs. A larger sample size was used compared to previous studies on validity for fitness tests in this population (de Oliveira Tavares et al. 2021). Additionally, a subgroup analysis explored potential variation factors that could affect validity, thereby enhancing the interpretation and generalizability of the findings. Finally, a standardized measurement protocol was applied for the KES test, and all assessments were performed by a single experienced evaluator to ensure standardization and minimize measurement variability. Future studies should evaluate the inter‐rater reliability of the 30s‐CST in adults with SMDs when administered by different healthcare professionals.

## 6 Implications for Physiotherapy Practice

The 30s‐CST is a practical, low‐cost, and accessible tool that may support the assessment of lower‐limb strength‐related functional performance in individuals with SMDs. The present findings suggest that it provides a feasible and pragmatic indicator related to lower‐limb MS in clinical settings where laboratory‐based assessments are not readily available. When interpreted alongside population‐specific reference values, the 30s‐CST may help identify individuals with reduced lower‐limb strength‐related functional performance and provide additional information to support routine physiotherapy assessment. Its simplicity and minimal equipment requirements make it particularly suitable for physiotherapy practice and multidisciplinary mental health services, where individuals with different SMD diagnoses are routinely managed and a common, feasible functional assessment may facilitate standardized physical evaluation.

## 7 Conclusions

In this study, the 30s‐CST demonstrated moderate criterion validity as an indicator of lower‐limb muscle strength in adults with SMDs, with higher concordance observed in the women subgroup. These findings suggest that the test may serve as a pragmatic screening tool for identifying individuals with reduced lower‐limb strength‐related functional performance. Given its simplicity, low cost, and minimal equipment requirements, the 30s‐CST represents a feasible option for assessing strength‐related functional limitations in mental health services where more complex methods are not readily available. Further longitudinal studies are warranted to evaluate its responsiveness, screening performance, and clinical utility for monitoring changes over time.

## Author Contributions

**Alvaro Lopez‐Moral:** principal investigator, contributed to conceptualization, methodology, investigation, formal analysis, writing – original draft preparation. **Diego Munguía‐Izquierdo:** contributed to funding acquisition, resources, writing – review and editing, supervision. **Javier Bueno‐Antequera:** contributed to investigation, writing – review and editing, supervision.

## Funding

This work was funded by Proyectos I + D + i 2020 (Ref. PID2020‐118262RB‐I00) from the Spanish Ministry of Science and Innovation. It was also supported by the CTS‐948 Research Group, Pablo de Olavide University, Junta de Andalucía, and FEDER Programme 2014–2020 (Ref. UPO‐1262802). ALM was supported by Pablo de Olavide University (Ref. PPI1803). JBA was supported by the Operational Programme FEDER Andalucía 2014–2020 (Ref. PAC2042) and by the Junta de Andalucía through the Consejería de Transformación Económica, Industria, Conocimiento y Universidades (Ref. POSTDOC_21_00059). Funding for open access publishing: Universidad Pablo de Olavide/CBUA. The funders were not involved in the design of the study, the collection, analysis, and interpretation of the data, the writing of the report, or the decision to publish.

## Consent

The authors assert that all procedures contributing to this work comply with the ethical standards of the relevant national and institutional committees on human experimentation and with the Helsinki Declaration of 1975, as revised in 2013. All procedures involving human subjects/patients were approved by the ethics committees of the Virgen Macarena and Virgen del Rocío University hospitals (1674‐N‐17). Consent for publication was obtained from all individual participants included in the study. Participants were informed about the nature, purpose, and potential implications of the research, and they provided written consent, acknowledging their understanding and agreement to the publication of the findings.

## Conflicts of Interest

The authors declare no conflicts of interest.

## Supporting information


Supporting Information S1

## Data Availability

The datasets generated and/or analyzed during the current study are available from the corresponding author on reasonable request.
