# Supplementary material for: Criterion Validity of the 30‐Second Chair Stand Test for Lower‐Limb Muscle Strength in Adults With Severe Mental Disorders: The PsychiActive Project
Source: Physiother Res Int. 2026 Sep 9;31(4):e70344. doi: 10.1002/pri.70344 (PMC13555177; doi:10.1002/pri.70344)
Supplement: Supplementary file 1 [file PRI-31-e70344-s001.docx]

**FIGURE S1**

*Bland-Altman Plots with limits of agreement (LOA) of* z*-scores for the 30s-CST and KES tests in adults with SMDs*

**Figure S1a**

Men (n = 63)

Differences z-score 30s-CST and KES

| Mean z-score 30s-CST and KES | dis_CS-LF |
| --- | --- |
| 0.038933 | -0.0677214 |
| -0.131351 | 0.272846 |
| 1.30212 | -0.585468 |
| -0.43687 | -0.12043 |
| 1.39934 | 1.56348 |
| 1.77486 | -2.87003 |
| -0.087368 | -0.484662 |
| 0.737378 | -0.460298 |
| 0.65681 | -0.299161 |
| 0.607768 | 0.803237 |
| 0.302644 | 0.0743998 |
| 1.06376 | 0.226028 |
| 1.12862 | 0.0963106 |
| -0.654783 | 0.315397 |
| -0.0539056 | 0.452727 |
| 0.955573 | 2.11626 |
| 0.389687 | 0.904627 |
| 0.845173 | 0.997969 |
| -0.850324 | -1.63692 |
| 0.128261 | 1.42748 |
| -0.0689475 | 0.14804 |
| 0.541114 | 0.601773 |
| 0.258544 | -0.841714 |
| -1.16011 | -0.347802 |
| -0.888476 | -0.556303 |
| 0.554956 | -0.764996 |
| 0.111159 | 0.122597 |
| -0.733963 | 0.473756 |
| -1.04282 | 0.0871651 |
| 1.46049 | 0.102109 |
| -1.2623 | -0.812962 |
| -0.65415 | -0.690184 |
| 0.765419 | -0.851151 |
| 0.534468 | 0.615066 |
| 0.570243 | -1.79988 |
| 0.102086 | -1.86788 |
| 0.792373 | -1.5746 |
| -0.125089 | 0.929864 |
| -0.661313 | 0.663228 |
| 0.00277223 | 0.00460012 |
| -1.10213 | 0.205771 |
| -0.90354 | -0.191404 |
| -0.15761 | -0.00940731 |
| -0.683531 | -0.966192 |
| 0.160418 | 0.0240795 |
| 0.126131 | 0.427426 |
| -1.27676 | 1.22459 |
| -1.13842 | 1.28267 |
| 0.380196 | -1.41979 |
| -0.427232 | 0.864609 |
| 0.474096 | 2.07489 |
| 0.164644 | -1.32346 |
| 0.0993209 | 1.48536 |
| -1.26149 | -0.479814 |
| -0.0119415 | 0.0340277 |
| -0.0816265 | -0.830917 |
| -2.13429 | -1.07761 |
| -0.831281 | 1.67271 |
| -0.435866 | -0.45721 |
| 0.380453 | 0.253552 |
| -0.760368 | -0.477747 |
| 0.793972 | 0.765601 |
| 0.66248 | 0.359041 |

| Mean z-score 30s-CST and KES | Mean | LOA upper | LOA lower |
| --- | --- | --- | --- |
| -4 | -0.0030546 | 1.92928 | -1.93539 |
| 4 | -0.0030546 | 1.92928 | -1.93539 |

*Note.* Results are stratified by sex (A: men, B: women), age (C: 18–40 years, D: 40–65 years) and BMI (E: < 30 kg/m², F: ≥ 30 kg/m²). The thick middle line represents the mean difference between the *z*-scores of the 30s-CST and KES tests, while the dashed lines represent the upper and lower 95% LOA (mean difference ± 1.96 *SD*).

**Figure S1b**

Women (n = 28)

Differences z-score 30s-CST and KES

| Mean z-score 30s-CST and KES | dis_CS-LF |
| --- | --- |
| 0.296129 | -0.190662 |
| -0.974927 | -0.0581245 |
| 1.8713 | -0.931433 |
| 1.02703 | 0.154714 |
| 1.14261 | 1.12834 |
| 0.989016 | -0.672846 |
| -0.541615 | -0.322354 |
| 0.182317 | 0.0369615 |
| 1.06714 | -0.226697 |
| -0.576838 | -1.75789 |
| 0.76209 | 1.28699 |
| -0.371875 | -0.360639 |
| -0.244202 | -1.51957 |
| -0.506046 | 0.510098 |
| -1.23872 | 0.168261 |
| 0.884925 | 0.740122 |
| 0.619408 | -0.536023 |
| -0.202642 | 0.204486 |
| 1.13116 | -0.0535406 |
| -1.08103 | 0.75648 |
| -0.680865 | 0.257341 |
| -0.792815 | 0.180045 |
| -1.29242 | 0.576866 |
| 0.25615 | 1.39528 |
| -0.738306 | 0.673421 |
| 0.619895 | 0.0653965 |
| -1.57397 | -0.0648224 |
| -1.27515 | -0.66246 |

| Mean z-score 30s-CST and KES | Mean | LOA upper | LOA lower |
| --- | --- | --- | --- |
| -4 | 0.0277762 | 1.48855 | -1.433 |
| 4 | 0.0277762 | 1.48855 | -1.433 |

**Figure S1c**

Age 18-40 years (n = 48)

Differences z-score 30s-CST and KES

| Mean z-score 30s-CST and KES | dis_CS-LF |
| --- | --- |
| 1.30212 | -0.585468 |
| 0.380453 | 0.253552 |
| 0.76209 | 1.28699 |
| 0.128261 | 1.42748 |
| 1.06714 | -0.226697 |
| 0.038933 | -0.0677214 |
| -0.831281 | 1.67271 |
| 0.955573 | 2.11626 |
| -0.680865 | 0.257341 |
| 0.607768 | 0.803237 |
| -0.760368 | -0.477747 |
| 0.25615 | 1.39528 |
| 0.845173 | 0.997969 |
| 0.474096 | 2.07489 |
| 1.02703 | 0.154714 |
| 1.12862 | 0.0963106 |
| 0.389687 | 0.904627 |
| -1.13842 | 1.28267 |
| 0.737378 | -0.460298 |
| -0.654783 | 0.315397 |
| 0.541114 | 0.601773 |
| 1.39934 | 1.56348 |
| -0.15761 | -0.00940731 |
| 0.0993209 | 1.48536 |
| -0.0119415 | 0.0340277 |
| -0.850324 | -1.63692 |
| 1.46049 | 0.102109 |
| -0.0816265 | -0.830917 |
| 1.13116 | -0.0535406 |
| -0.0539056 | 0.452727 |
| 0.258544 | -0.841714 |
| 0.554956 | -0.764996 |
| 0.126131 | 0.427426 |
| 0.380196 | -1.41979 |
| 1.8713 | -0.931433 |
| -0.43687 | -0.12043 |
| -0.125089 | 0.929864 |
| -1.16011 | -0.347802 |
| 0.534468 | 0.615066 |
| 0.619895 | 0.0653965 |
| -0.087368 | -0.484662 |
| 0.65681 | -0.299161 |
| 0.792373 | -1.5746 |
| 0.111159 | 0.122597 |
| 0.160418 | 0.0240795 |
| -0.435866 | -0.45721 |
| 0.884925 | 0.740122 |
| -1.27515 | -0.66246 |

| Mean z-score 30s-CST and KES | Mean | LOA upper | LOA lower |
| --- | --- | --- | --- |
| -4 | 0.207302 | 1.98644 | -1.57184 |
| 4 | 0.207302 | 1.98644 | -1.57184 |

**Figure S1d**

Age 40-65 years (n = 43)

Differences z-score 30s-CST and KES

| Mean z-score 30s-CST and KES | dis_CS-LF |
| --- | --- |
| 0.302644 | 0.0743998 |
| -1.10213 | 0.205771 |
| -0.90354 | -0.191404 |
| 1.06376 | 0.226028 |
| -0.427232 | 0.864609 |
| 0.164644 | -1.32346 |
| -1.26149 | -0.479814 |
| -0.131351 | 0.272846 |
| 0.296129 | -0.190662 |
| -1.57397 | -0.0648224 |
| -0.661313 | 0.663228 |
| 0.00277223 | 0.00460012 |
| 0.793972 | 0.765601 |
| -0.974927 | -0.0581245 |
| -0.0689475 | 0.14804 |
| 0.570243 | -1.79988 |
| -2.13429 | -1.07761 |
| 0.989016 | -0.672846 |
| -0.576838 | -1.75789 |
| -0.371875 | -0.360639 |
| -1.23872 | 0.168261 |
| -0.888476 | -0.556303 |
| 0.765419 | -0.851151 |
| 1.14261 | 1.12834 |
| 1.77486 | -2.87003 |
| -1.04282 | 0.0871651 |
| -1.29242 | 0.576866 |
| -0.244202 | -1.51957 |
| 0.66248 | 0.359041 |
| 0.182317 | 0.0369615 |
| -1.08103 | 0.75648 |
| -1.2623 | -0.812962 |
| -0.541615 | -0.322354 |
| -1.27676 | 1.22459 |
| 0.619408 | -0.536023 |
| -0.738306 | 0.673421 |
| -0.733963 | 0.473756 |
| -0.683531 | -0.966192 |
| 0.102086 | -1.86788 |
| -0.202642 | 0.204486 |
| -0.792815 | 0.180045 |
| -0.65415 | -0.690184 |
| -0.506046 | 0.510098 |

| Mean z-score 30s-CST and KES | Mean | LOA upper | LOA lower |
| --- | --- | --- | --- |
| -4 | -0.217795 | 1.5051 | -1.94069 |
| 4 | -0.217795 | 1.5051 | -1.94069 |

**Figure S1e**

BMI < 30 kg/m² (n = 44)

Differences z-score 30s-CST and KES

| Mean z-score 30s-CST and KES | dis_CS-LF |
| --- | --- |
| 0.038933 | -0.0677214 |
| -0.131351 | 0.272846 |
| 0.296129 | -0.190662 |
| -0.974927 | -0.0581245 |
| 1.8713 | -0.931433 |
| 1.02703 | 0.154714 |
| 1.30212 | -0.585468 |
| -0.43687 | -0.12043 |
| 1.14261 | 1.12834 |
| 1.39934 | 1.56348 |
| 0.989016 | -0.672846 |
| 1.77486 | -2.87003 |
| -0.087368 | -0.484662 |
| 0.737378 | -0.460298 |
| 0.65681 | -0.299161 |
| 0.607768 | 0.803237 |
| -0.541615 | -0.322354 |
| 0.302644 | 0.0743998 |
| 1.06376 | 0.226028 |
| 1.12862 | 0.0963106 |
| -0.654783 | 0.315397 |
| -0.0539056 | 0.452727 |
| 0.955573 | 2.11626 |
| 0.389687 | 0.904627 |
| 0.182317 | 0.0369615 |
| 0.845173 | 0.997969 |
| -0.850324 | -1.63692 |
| 1.06714 | -0.226697 |
| -0.576838 | -1.75789 |
| 0.128261 | 1.42748 |
| -0.0689475 | 0.14804 |
| 0.541114 | 0.601773 |
| 0.258544 | -0.841714 |
| 0.76209 | 1.28699 |
| -1.16011 | -0.347802 |
| -0.888476 | -0.556303 |
| 0.554956 | -0.764996 |
| -0.371875 | -0.360639 |
| 0.111159 | 0.122597 |
| -0.244202 | -1.51957 |
| -0.733963 | 0.473756 |
| -0.506046 | 0.510098 |
| -1.04282 | 0.0871651 |
| 1.46049 | 0.102109 |

| Mean z-score 30s-CST and KES | Mean | LOA upper | LOA lower |
| --- | --- | --- | --- |
| -4 | -0.026646 | 1.77354 | -1.82684 |
| 4 | -0.026646 | 1.77354 | -1.82684 |

**Figure S1f**

BMI ≥ 30 kg/m² (n = 47)

Differences z-score 30s-CST and KES

| Mean z-score 30s-CST and KES | dis_CS-LF |
| --- | --- |
| -1.2623 | -0.812962 |
| -0.65415 | -0.690184 |
| 0.765419 | -0.851151 |
| 0.534468 | 0.615066 |
| 0.570243 | -1.79988 |
| 0.102086 | -1.86788 |
| 0.792373 | -1.5746 |
| -0.125089 | 0.929864 |
| -0.661313 | 0.663228 |
| -1.23872 | 0.168261 |
| 0.00277223 | 0.00460012 |
| -1.10213 | 0.205771 |
| -0.90354 | -0.191404 |
| -0.15761 | -0.00940731 |
| -0.683531 | -0.966192 |
| 0.160418 | 0.0240795 |
| 0.884925 | 0.740122 |
| 0.126131 | 0.427426 |
| 0.619408 | -0.536023 |
| -0.202642 | 0.204486 |
| -1.27676 | 1.22459 |
| -1.13842 | 1.28267 |
| 1.13116 | -0.0535406 |
| 0.380196 | -1.41979 |
| -0.427232 | 0.864609 |
| -1.08103 | 0.75648 |
| 0.474096 | 2.07489 |
| 0.164644 | -1.32346 |
| 0.0993209 | 1.48536 |
| -0.680865 | 0.257341 |
| -1.26149 | -0.479814 |
| -0.792815 | 0.180045 |
| -0.0119415 | 0.0340277 |
| -0.0816265 | -0.830917 |
| -1.29242 | 0.576866 |
| -2.13429 | -1.07761 |
| -0.831281 | 1.67271 |
| -0.435866 | -0.45721 |
| 0.380453 | 0.253552 |
| -0.760368 | -0.477747 |
| 0.793972 | 0.765601 |
| 0.66248 | 0.359041 |
| 0.25615 | 1.39528 |
| -0.738306 | 0.673421 |
| 0.619895 | 0.0653965 |
| -1.57397 | -0.0648224 |
| -1.27515 | -0.66246 |

| Mean z-score 30s-CST and KES | Mean | LOA upper | LOA lower |
| --- | --- | --- | --- |
| -4 | 0.0373982 | 1.84005 | -1.76526 |
| 4 | 0.0373982 | 1.84005 | -1.76526 |
